# Characteristic Manifestations regarding Ultrasound Biomicroscopy Morphological Data in the Diagnosis of Acute Angle Closure Secondary to Lens Subluxation

**DOI:** 10.1155/2019/7472195

**Published:** 2019-06-25

**Authors:** Fenglei Wang, Dabo Wang, Ling Wang

**Affiliations:** Department of Ophthalmology, The Affiliated Hospital of Qingdao University, Qingdao, China

## Abstract

**Purpose:**

To investigate the mechanisms underlying the occurrence of acute angle closure (AAC) and to further explore the sensitive indicators for clinical diagnosis of acute angle closure secondary to lens subluxation (AACSLS) through qualitative and quantitative analysis of ultrasound biomicroscopy (UBM) imaging features of eyes with AAC to provide a theoretical basis for the selection of treatment schemes.

**Methods:**

A retrospective analysis was conducted from 2013 to 2018 on 160 eyes (160 patients) with uniocular acute angle closure crisis (AACC) complicated by cataract. The case group consisted of 29 eyes (29 patients) with lens subluxation and the control group consisted of 131 eyes (131 patients) without lens subluxation. Before the operation, computer optometry, best corrected visual acuity, intraocular pressure, slit lamp, gonioscopy, preset lens, A-mode ultrasonography, and UBM were performed. All the enrolled subjects underwent cataract surgery with or without other operations. The pupil was fully dilated, and the position of the lens was recorded before the operation. The zonular rupture and lens subluxation were further confirmed during operation. SPSS version 20.0 was used to analyze UBM imaging data from the lens subluxation group and non-lens subluxation group.

**Results:**

The iris span (IS) value in the whole quadrant of the lens subluxation group was significantly higher than that of the non-lens subluxation group (P=0.033, 95%CI 0.01 to 0.31). The iris lens angle (ILA) in the lens subluxation group was significantly lower than that in the non-lens subluxation group in the upper, lower, nasal, temporal, and whole quadrants (P<0.001, 95%CI -8.79 to -2.78; P=0.001, 95%CI -8.36 to -2.27; P<0.001, 95%CI -9.85 to -4.98; P=0.015, 95%CI -6.67 to -0.72; P<0.001, 95%CI -8.74 to -5.83, respectively). However, the ILA of the maximum difference among the four quadrants in the lens subluxation group was significantly higher than that in the non-lens subluxation group (P<0.001, 95%CI 4.74 to 9.86). The ILA and iris lens contact distance (ILCD) showed significant negative correlations in both the lens subluxation group and non-lens subluxation group (Y=20.984-7.251X, R=0.520, and P<0.001; Y=19.923-3.491X, R=0.256, and P<0.001, respectively). The risk ratio of lens subluxation in exposed eyes with ILA=0 in one quadrant at least was significantly higher than that in nonexposed eyes without ILA=0 in all quadrants (*X*^*2*^=87.859, P<0.001, and odds ratio (OR)=79.200, 95% CI 23.063 to 271.983). The risk ratio of zonular rupture in exposed quadrants with ILA=0 was significantly lower than that in nonexposed eyes without ILA=0 (*X*^*2*^=33.884, P<0.001, OR=0.122, and 95% CI 0.053 to 0.278). The risk ratio of zonular rupture in exposed quadrants with nonforward convexity of iris was significantly lower than that in nonexposed quadrants with forward convexity of iris (*X*^*2*^=6.413, P=0.011, and OR=0.381; 95% CI 0.176 to 0.825).

**Conclusions:**

ILA=0 and nonforward convexity of iris as UBM sensitive and characteristic indicators for screening lens subluxation and zonular rupture can provide new ideas and hints for clinical diagnosis of acute angle closure secondary to lens subluxation.

## 1. Introduction

Glaucoma is the second leading cause of blindness in the world [1, 2]. Glaucomatous optic neuropathy is expected to occur in approximately 80 million patients worldwide by 2020 [3, 4]. Population-based epidemiological studies have found that the incidence of angle closure glaucoma in the East Asian population is much higher than that in European and African populations [5]. Eighty-seven percent of ACG patients live in Asia, and two million Chinese patients suffer from blindness in at least one eye due to primary angle closure glaucoma (PACG) [6]. By 2020, it is estimated that, in more than half of patients with binocular blindness, the cause will be PACG [7].

The 2016 Primary Angle Closure Preferred Practice Pattern (PPP) Guidelines issued by the US [8] point out that acute angle closure crisis (AACC) is often accompanied by acute anterior chamber angle blockage and rapid rise in intraocular pressure (IOP). The treatment for AACC-affected eyes is mainly external trabeculectomy and can be combined with cataract surgery. A large number of studies have found that lens extraction can significantly widen the anterior chamber angle (narrow chamber angle, closable chamber angle, and angle closure glaucoma) [9–14].

Ultrasound biomicroscopy (UBM), as a noninvasive and high-resolution in vivo anterior segment imaging technique, has been proven to have great advantages in evaluating anterior chamber angle structure [15]. UBM can image and evaluate the morphological structure of the anterior segment of the eye including the ciliary body, the zonule, and the anterior surface of the lens after iris covering, which cannot be observed by routine ophthalmic examination. In addition, quantitative and qualitative analysis of pathophysiological changes of the anterior segment structure can be conducted simultaneously [16, 17].

Imaging diagnosis of lens subluxation has long been an important clinical research topic; however, there are no clear diagnostic criteria worldwide. The zonule is deeply located with fibrous distribution and with different degrees of sparseness in each quadrant. The attachment positions of the lens and ciliary process are often variable. Therefore, it is difficult to directly observe the morphological characteristics of the occult zonular defects. Although UBM can make an assessment of the relative position of the lens in the center of the eyeball, there is a prominent learning curve in the diagnosis for zonular ruptures by UBM [18]. To our knowledge, there are few related clinical studies available on sensitive indicators for diagnosis of lens subluxation. Yaguchi et al. [19] indirectly determined the degree of relaxation of the zonule by observing the degree of lens displacement during anterior capsular tear before cataract surgery. This can provide clinical guidance for the selection of surgical equipment and surgical methods.

In this study, we performed analysis on the qualitative and quantitative data of UBM from patients with definite diagnosis of acute angle closure secondary to lens subluxation (AACSLS) and primary acute angle closure (PAAC) after mydriasis and during cataract surgery. In addition, we explored the sensitive indicators for UBM imaging diagnosis of AACSLS and analyzed the correlation among various indicators to advance new ideas for clinical diagnosis of AACSLS, thus providing a theoretical basis for selection of treatment schemes.

## 2. Materials and Methods

### 2.1. Materials

#### 2.1.1. Study Population

Data were collected from 160 patients with uniocular acute angle closure crisis (AACC) who were admitted to the Affiliated Hospital of Qingdao University from October 2013 to October 2018. Only 160 affected eyes (160 patients) were selected as clinical research cases, with 133 from females (133 eyes, 83.12%) and 27 from males (27 eyes, 16.88%), who had an average age of 66.54±8.44 years (Table 1).

The case group consisted of 29 eyes from 29 patients (23 females and 6 males) with lens subluxation (LS), with an average age of 68.55±7.85 years, among which 14 were right eyes and 15 were left eyes. The control group consisted of 131 non-lens subluxation (NLS) eyes from 131 patients (110 females and 21 males), with an average age of 66.09±8.52 years, among which 65 were right eyes and 66 were left eyes (Table 1). After admission, all patients signed the informed consent form for admission, the informed consent form for operation, and the patient information sheet for matters regarding participation in the clinical study. All the processes of the study conformed to the Declaration of Helsinki and were reviewed and approved by the Ethics Committee of the Affiliated Hospital of Qingdao University. This study was purely clinical scientific research and did not involve any commercial activities.

#### 2.1.2. Inclusion and Exclusion Criteria

The inclusion and exclusion criteria included the following:

1. There should not be any major underlying disease requiring medical or surgical intervention (excluding patients with hypertension, diabetes, dialysis for renal failure, immune diseases requiring long-term oral hormone treatment, and long-term chemotherapy after surgery for malignant tumor)
2. There should be uniocular acute angle closure glaucoma, with the time of onset less than 5 days. Before admission, neither of the eyes has received medication, laser, or surgical intervention (including anterior chamber puncture treatment). The patient should not have any history of ocular trauma in either eye
3. Ophthalmic examination does not reveal any ophthalmic diseases affecting the chamber angle like iris root detachment, anterior chamber angle recession, space occupying lesions in the anterior and posterior ocular segments, suprachoroidal effusion (ciliary body or choroidal detachment), retinal detachment, acute or old uveitis, etc.
4. Typical characteristics of AACC [8] are present during disease onset, such as
  1. Presence of at least one of the following symptoms: periocular pain, headache, nausea and/or vomiting, decreased visual acuity, and/or a history of intermittent iridization attacks
  2. IOP ≥ 21 mmHg (measured by Goldmann applanation tonometer);
  3. The contact range of angle trabecular observed under gonioscope exceeding 180
  4. Presence of at least four abnormal eye signs observed under slit lamp: ciliary congestion, corneal endothelial edema, fixed medium-sized pupil, glaucomatous fleck, and shallow peripheral anterior chamber
5. The clinical features of the enrolled patients suggest the possibility of lens-induced acute angle closure (lens dilation, advancement of iris diaphragm in the lens, lens shaking, iridodonesis, etc.), mild or higher degree of clouding in the lens [21, 22], and the patients' strong desire to improve visual function
6. Cataract lens extraction was performed for all patients enrolled. Extracapsular lens extraction (ECCE), phacoemulsification (Phaco), and posterior-approach lensectomy were selected according to the preoperative examinations and intraoperative conditions. External trabeculectomy, simple peripheral iridotomy, anterior chamber angle goniosynechialysis, intraocular lens implantation, or posterior-approach vitrectomy might be combined during the operation.
7. Excluding patients with allergy to the mydriatic agent (compound tropicamide eye drops: eye drops containing 0.5% tropicamide and 0.5% phenylephrine hydrochloride) and surface anesthetic agent (oxybuprocaine hydrochloride eye drops: 0.4% oxybuprocaine solution, 20mL:80mg)
8. Patients with poor image clarity in UBM, A-Scan, and other imaging examinations that cannot clearly distinguish the anatomical structures and morphological characteristics are excluded
9. Patients with incomplete clinical data are excluded, as this makes later data statistics and analysis extremely difficult.

### 2.2. Methods

#### 2.2.1. General Ophthalmic Examination

Patients were asked about their medical history and it was recorded in detail for all subjects after admission.

Ophthalmic examination included computer optometry (Topcon Ltd., Model KR-8900, Japan), best corrected visual acuity (Topcon Ltd., Model CV-5000, Japan), intraocular pressure (Goldmann applanation tonometer), slit lamp, and related examinations (preset lens, gonioscopy) (Haag-Streit Ltd., Model BM 900, Switzerland).

A-Scan (Quantel Medical Ltd., Model Aviso, France) was carried out to measure axial length (AL), central anterior chamber depth (CACD), and lens thickness (LT). The parameters of lens position should be used in the study, which can be obtained indirectly through the above data calculation: lens-axial length factor (LAF)=LT/AL *∗* 10; lens position (LP)=CACD + 1/2LT; relative lens position (RLP)=LP/AL *∗* 10 [23–26].

Ultrasound biomicroscopy (Suoer Electronic Ltd., Model SW3200L, China) was carried out to measure relevant parameters [23, 27] (see details below).

#### 2.2.2. UBM Imaging Quantitative Data Acquisition Method

(1) Angle opening distance (angle opening distance at 500*μ*m from scleral spur, AOD 500) [26]: The specific measurement method was to start at a point 500*μ*m from the scleral spur along the corneal endothelium surface and make a line perpendicular to the corneal endothelium through this point. The perpendicular line intersected with the anterior iris surface. This vertical line was AOD 500. This parameter can indirectly reflect the degree of chamber angle opening.

(2) Trabecular iris angle (TIA) [28]: The clinical TIA value was consistent with the anterior chamber angle of 500*μ*m (anterior chamber angle at 500*μ*m from scleral spur, ACA 500). The specific measurement method was to make a triangle with AOD 500 as the base and the recess at the iris root as the vertex, and the included angle of the vertex was TIA. This parameter can indirectly reflect the degree of chamber angle opening.

(3) Iris convexity (IC) [29–31]: Iris convexity is the curvature of the posterior surface of the iris and is indirectly expressed by the length of the vertical line from the most protruding position of the iris to the line connecting the iris root and the iris apex [20, 28, 32, 33]. A positive value of IC represented forward convexity of the iris, and a negative value represented posterior iris bombe. For iris with both anterior and posterior bombe, the direction with greater bombe was taken.

(4) Iris span (IS): It is the straight-line distance from the attachment point of the root of the posterior iris surface to the iris apex (the iris apex is the midpoint of the iris lens contact distance (ILCD)). This parameter can directly reflect the average distance that the iris extends to the central part of the eyeball and indirectly reflects the size of the pupil.

(5) Iris lens angle (ILA) [28]: The specific measurement method was to take the contact point between the posterior iris surface and the anterior lens surface as the vertex, and two sides along this vertex were tangent lines of the posterior iris surface and the anterior lens surface, respectively. The included angle formed was ILA. This parameter can directly reflect the relative position of the lens and central iris and indirectly reflect the degree of attachment and detachment of the lens and iris.

(6) Iris lens contact distance (ILCD) [28]: It is the line between the contact points of the anterior and posterior iris surfaces and the anterior lens surface. This parameter can directly reflect the degree of attachment and detachment of the lens and iris and indirectly reflect the relative positions of the two.

(7) Iris ciliary processes angle (ICPA): It is the angle between the root of the posterior surface of the iris and the anterior surface of the ciliary process. This parameter can directly reflect the positional relationship between ciliary process and iris root.

(8) Limbus ciliary body angle (LCBA): The two sides of the angle are, respectively, the extension line of the connection line from the central point of the ciliary process to the central point of the ciliary body basement and the extension of the connection line between the central point of limbal thickness and the central point of one-third thickness of the lateral part of the cornea along the direction of the long axis of the ciliary body. The two sides can reflect the average trend of ciliary body and corneal limbus, and this included angle can directly reflect the positional relationship and degree of separation (pronation or supination) between ciliary body and corneal limbus. It can also reflect the relative position of the whole ciliary body inside the eyeball.

**Figure 1:**
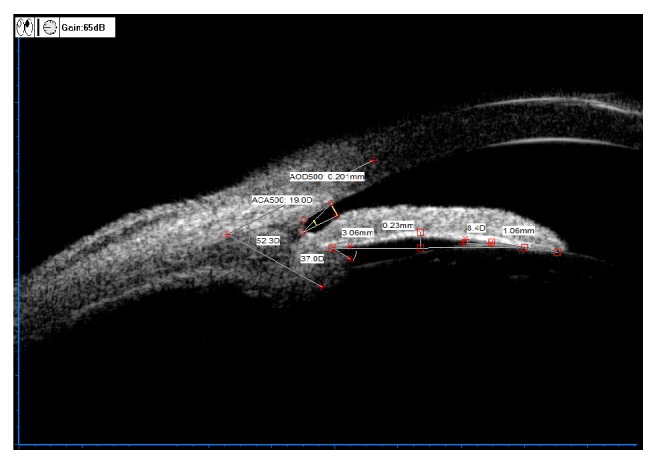
Standardized collection for quantitative data of ocular UBM image.

Figure 1 is a local image of the nasal quadrant of the left eye of a UBM scanning case. The manual labeling and calculation of quantitative data were completed by using the UBM's own labeling software and then directly obtaining output (the specific output data were as follows: AOD500=0.201mm; ACA500 (TIA)=19.0D; IC=0.23mm; IS=3.06mm; ILA=8.4D; ILCD=1.06mm; LCPA=37.0D; LCBA=52.3D).

### 2.3. Statistical Analysis

SPSS version 20.0 statistical software (SPSS, Inc., Chicago, IL) was used for data analysis. Independent samples* t*-test was used to compare and analyze UBM imaging quantitative data, demographic characteristics (ages and eyes), and standard clinical data (onset time, intraocular pressure, best corrected visual acuity, and spherical lens diopter) of the lens subluxation group and the non-lens subluxation group. The quantitative data ILA and ILCD were subjected to univariate linear regression analysis, and the regression formula and R value were calculated to create univariate scatter plots. Chi-square test was used to compare the gender proportions of the two groups and the proportions of lens subluxation and zonular rupture under different exposure factors, and the Pearson Chi-square value (*χ*^2^) was calculated. Simultaneously, the odds ratio (OR) was calculated and output obtained. P<0.05 indicated statistically significant difference.

## 3. Results

### 3.1. Population Characteristics and Clinical Data

There was no significant difference in gender, age, and affected eye between the lens subluxation group and the non-lens subluxation group (P=0.544; P=0.156; P=0.896, respectively); however, the clinical onset time in the lens subluxation group was significantly shorter than that in the non-lens subluxation group (P=0.028) (Table 1). In the lens subluxation group, the intraocular pressure was 37.10±17.33 mmHg, best corrected visual acuity was 0.17±0.26, spherical diopter was 0.82±2.02D, and cylindrical diopter was -0.17±1.10D. In the non-lens subluxation group, the intraocular pressure was 34.91±15.73 mmHg, the best corrected visual acuity was 0.22±0.26, the spherical diopter was 0.83±2.00D, and the cylindrical diopter was -0.18±1.06D. There was no significant difference between the groups in the four parameters (P=0.505; P=0.420; P=0.832; P=0.945, respectively).

### 3.2. Interpretation of Image

**Figure 2:**
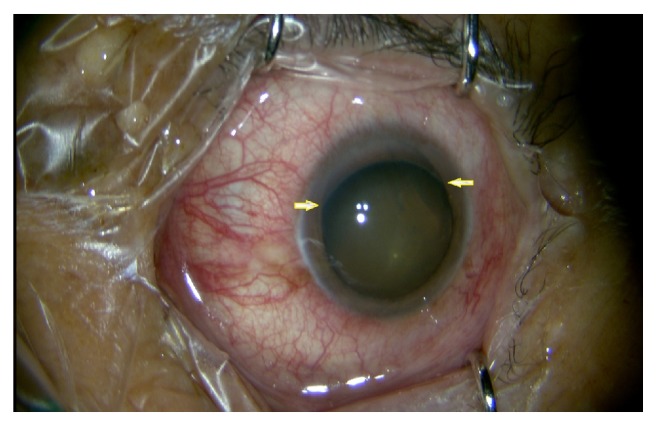
Lens subluxation and zonular rupture observed in the superior and nasal quadrants of right eye before surgery (yellow arrows).

Figure 2 shows the eye (right eye) of a patient with AAC scheduled to undergo cataract combined with glaucoma surgery. After sufficient mydriasis before surgery, it was found that the lens was displaced to the temporal side and below, and the zonule disappeared on the nasal side and above. Data collection was as follows: dislocation range of 10 o'clock to 3 o'clock; quadrant of zonular rupture: nasal side and upper part. The results were recorded, and the data were analyzed.

**Figure 3:**
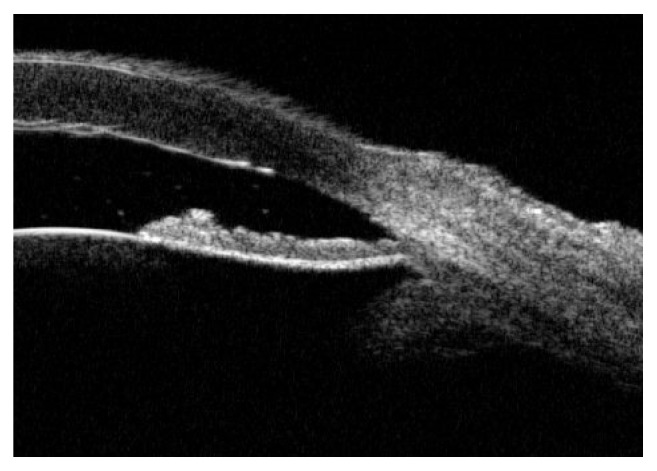
Lens subluxation with ILA=0° and backward convexity of central iris.

Figure 3 shows ILA=0 in UBM images of AAC eyes diagnosed with lens subluxation during operation, and the iris morphology was backward convexity of central iris accompanied by forward convexity of root iris.

**Figure 4:**
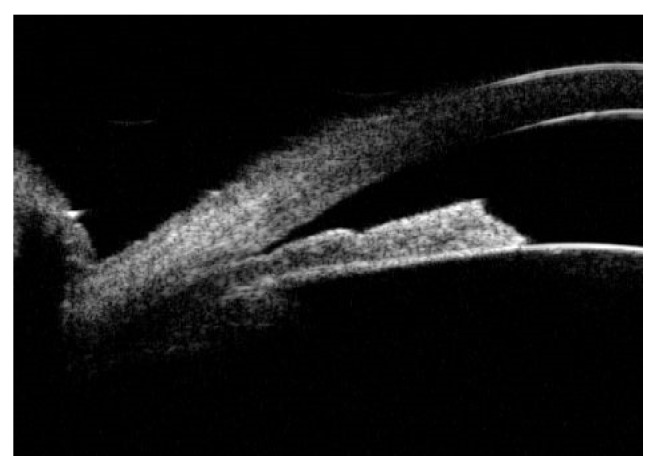
Lens subluxation with ILA=0° and flat iris.

Figure 4 shows ILA=0 in UBM images of AAC eyes diagnosed with lens subluxation during operation, and the iris morphology was flat.

**Figure 5:**
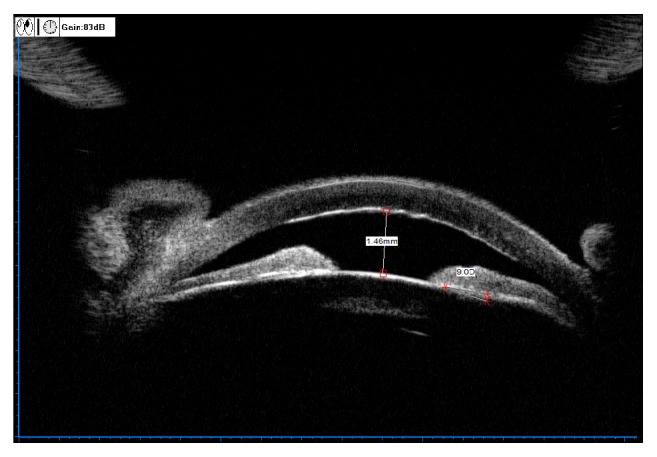
ILA=0° and flat iris in the superior quadrant of left eye, with lens subluxation and zonular rupture on the other side.

Figure 5 shows ILA=0 in the upper quadrant in the UBM panoramic scan image of an AAC eye (left eye) diagnosed with lens subluxation during operation, and the iris morphology was flat. ILA=9D in the lower quadrant, and the iris morphology was forward convexity of the whole iris.

**Figure 6:**
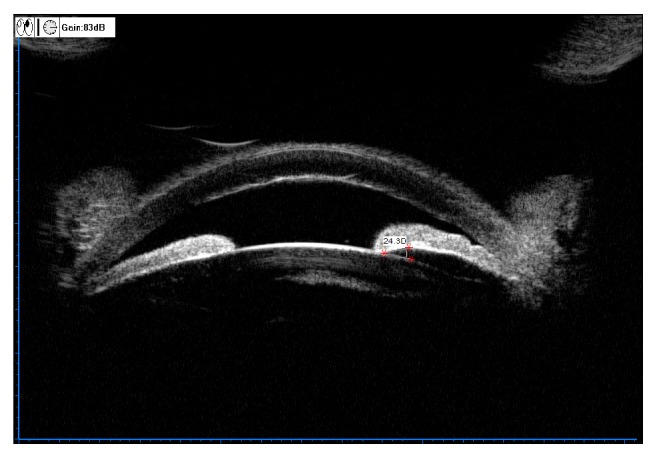
ILA=0° and slight forward convexity of iris in the temporal quadrant of left eye in the same patient (Figure 5), with lens subluxation and zonular rupture on the other side.

Figure 6 shows ILA=0 in the temporal quadrant in the UBM panoramic scan image of the same affected eye of the same patient as in Figure 5, and the iris morphology was forward convexity of the whole iris. ILA=24.3D in the nasal quadrant, and the iris morphology was forward convexity of the whole iris.

Both figures show that the iris diaphragm of the lens moved forward significantly, and the lens showed a “seesaw” change, with one equatorial portion elevated and one equatorial portion lowered, which might be related to uneven traction force at the equatorial portion of the lens caused by lens subluxation and zonular rupture in parts of the quadrants.

**Figure 7:**
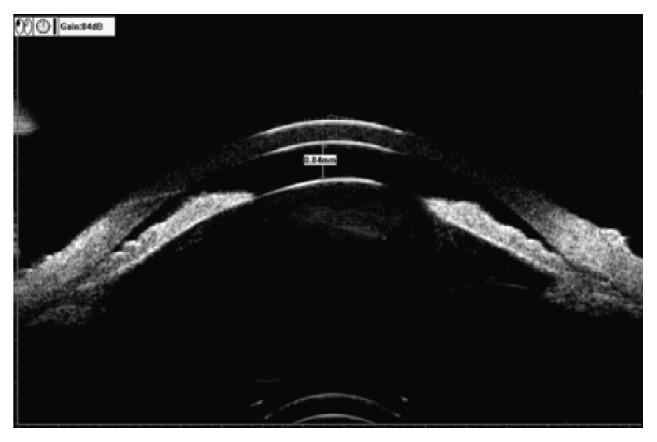
ILA=0° and basal backward convexity of iris in the superior quadrant of right eye from Luo's study, with significant lens subluxation and zonular rupture on the other side [20].

Figure 7 shows significant lens subluxation with the same characteristic UBM image changes as in Figures 5 and 6 [34] (image from the study by Luo et al.).

### 3.3. Comparative Analysis of UBM Imaging Quantitative Data

There were no significant differences in the maximum difference values in AOD500 upper, lower, nasal, temporal, whole, and four quadrants between the lens subluxation group and the non-lens subluxation group (P=0.662; P=0.282; P=0.890; P=0.638; P=0.502; P=0.428, respectively).

There were no significant differences in the maximum difference values of TIA in the upper, lower, nasal, temporal, whole, and four quadrants between the two groups (P=0.735; P=0.263; P=0.923; P=0.761; P=0.551; P=0.522, respectively).

There were no significant differences in the maximum difference values of IC in the upper, lower, nasal, temporal, whole, and four quadrants between the two groups (P=0.447; P=0.726; P=0.953; P=0.296; P=0.196; P=0.396, respectively).

There were no significant differences in the maximum difference values of IS in the upper, lower, nasal, temporal, whole, and four quadrants between the two groups (P=0.478; P=0.115; P=0.298; P=0.585; P=0.253, respectively). However, the IS value in the whole quadrant of the lens subluxation group was significantly higher than that of the non-lens subluxation group (P=0.033, 95%CI 0.01 to 0.31).

The ILA values in the upper, lower, nasal, temporal, and whole quadrants in the lens subluxation group were significantly lower than those in the non-lens subluxation group (P<0.001, 95%CI -8.79 to -2.78; P=0.001, 95%CI -8.36 to -2.27; P<0.001, 95%CI -9.85 to -4.98; P=0.015, 95%CI -6.67 to -0.72; P<0.001, 95%CI -8.74 to -5.83, respectively). However, the maximum difference value of ILA among the four quadrants in the lens subluxation group was significantly higher than that in the non-lens subluxation group (P<0.001, 95%CI 4.74 to 9.86).

There were no significant differences in the maximum difference values of ILCD in the upper, lower, nasal, temporal, whole, and four quadrants between the two groups (P=0.301; P=0.157; P=0.091; P=0.115, respectively); however, the ILCD values in the nasal side and whole quadrants of the lens subluxation group were significantly greater than those of the non-lens subluxation group (P=0.017, 95%CI 0.06 to 0.54; P=0.001, 95%CI 0.07 to 0.29, respectively).

There were no significant differences in the maximum difference values of ICPA in the upper, lower, nasal, temporal, whole, and four quadrants between the two groups (P=0.202; P=0.508; P=0.945; P=0.139; P=0.195, respectively); however, the ICPA in the lower quadrant of the lens subluxation group was significantly greater than that in the non-lens subluxation group (P=0.044, 95%CI 0.25 to 18.29).

There were no significant differences in the maximum difference values of LCBA in the upper, lower, nasal, temporal, whole, and four quadrants between the two groups (P=0.421; P=0.266; P=0.973; P=0.430; P=0.235; P=0.839, respectively).

There were no significant differences in AL, CACD, LP, or RLP between the two groups (P=0.966; P=0.393; P=0.149; P=0.183, respectively); however, LT and LAF in the lens subluxation group were significantly higher than those in the non-lens subluxation group (P=0.022, 95%CI 0.04 to 0.50; P=0.041, 95%CI 0.00 to 0.23, respectively) (Table 2).

### 3.4. Univariate Linear Regression Analysis and Scatter Diagram

**Figure 8:**
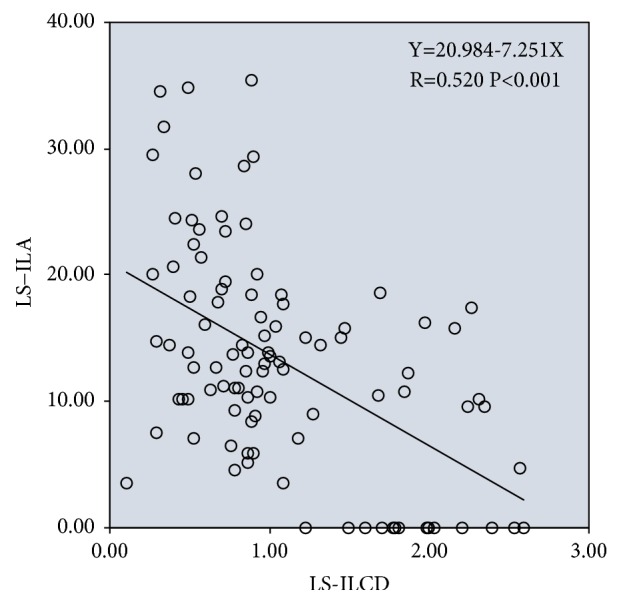
Linear regression between ILA and ILCD in AAC eyes with lens subluxation (LS).

Univariate linear regression analysis showed that ILA had a significant negative correlation with ILCD in the lens subluxation group (linear regression equation: Y=20.984-7.251X, R=0.520, and P<0.001); i.e., the smaller the angle of ILA, the greater the length of ILCD (Figure 8).

**Figure 9:**
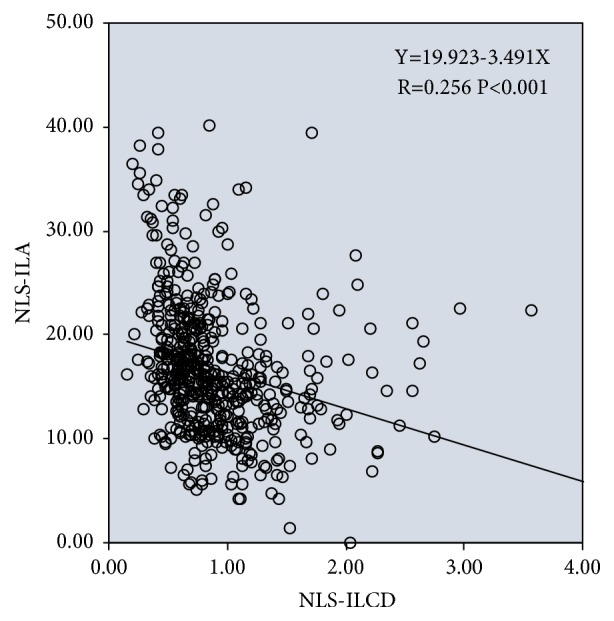
Linear regression between ILA and ILCD in AAC eyes with non-lens subluxation (NLS).

Univariate linear regression analysis showed that ILA had a significant negative correlation with ILCD in the non-lens subluxation group (linear regression equation: Y=19.923-3.491X, R=0.256, and P<0.001); i.e., the smaller the angle of NLS-ILA, the greater the length of NLS-ILCD (Figure 9).

### 3.5. Determination of Odds Ratios and Chi-Square Analysis

ILA=0 in at least one quadrant of the eye with AAC was taken as the risk exposure factor, and the proportion of eyes with lens subluxation and without lens subluxation in the exposure and nonexposure states was analyzed. The results were interpreted as follows (Table 3).

The risk ratio of lens subluxation in eyes in the exposure group with ILA=0 in at least one quadrant was significantly higher than that in eyes in the nonexposure group without ILA=0 in any of the quadrants (*χ*^2^=87.859, P<0.001; OR=79.200; 95%CI 23.063 to 271.983).

ILA=0 in the quadrants of the eye with AAC was regarded as the risk exposure factor, and the proportions of quadrants with and without zonular rupture in the exposure and nonexposure states were analyzed. The results were interpreted as follows (Table 4).

The risk ratio of zonular rupture in the quadrants with ILA=0 in the exposure group was significantly lower than that in the quadrants without ILA=0 in the nonexposure group (*χ*^2^=33.884, P<0.001; OR=0.122; 95%CI 0.053 to 0.278). ILA=0 was a protective factor for zonular rupture in the quadrant.

Nonforward convexity of iris in at least one quadrant of the eye with AAC was taken as the risk exposure factor to analyze the proportion of eyes with and without lens subluxation in the exposure and nonexposure states. The results were interpreted as follows (Table 5).

The risk ratio of lens subluxation in eyes in the exposure group with nonforward convexity of iris in at least one quadrant and in eyes in the nonexposure group without nonforward convexity of iris in any of the quadrants was not significantly different (*χ*^2^=1.426, P=0.232; OR=0.597; 95%CI 0.255 to 1.400).

Nonforward convexity of iris in the quadrants of the eye with AAC was taken as the risk exposure factor, and the proportions of quadrants with and without zonular rupture in the exposure and nonexposure states were analyzed. The results were interpreted as follows (Table 6).

The risk ratio of zonular rupture in the exposure group quadrants with nonforward convexity of iris was significantly lower than that in the nonexposure group quadrants without nonforward convexity of iris (*χ*^2^=6.413, P=0.011; OR=0.381; 95%CI 0.176 to 0.825). Nonforward convexity of iris was a protective factor for zonular rupture.

## 4. Discussion

The occurrence of lens subluxation is occult, which makes its clinical diagnosis difficult. Imaging examinations (UBM, A-scan, and anterior segment optical coherence tomography (AS-OCT)) cannot confirm the diagnosis. The main ocular signs are shallower anterior chamber depth, uneven anterior chamber depth among the quadrants, great difference in anterior chamber depth in the center of each eye, iridodonesis, lens shaking, anterior displacement of iris lens diaphragm, etc. The clinical diagnosis is based on the fact that after the pupil is fully dilated the rupture and disappearance of the zonule at the equator and dislocation of the lens can be seen in some quadrants. Clinical observation found that the onset of AAC is often accompanied by lens subluxation; however, mydriasis may lead to iris accumulation in the angle, aggravating the acute angle closure. Therefore, it is difficult to make a definite diagnosis and conduct prospective cohort studies. Hence, we retrospectively selected patients who needed cataract surgery (combined/not combined with other surgical methods) to solve AAC. Preoperative mydriasis and intraoperative observation can confirm whether the patient has lens subluxation and zonular rupture. Detailed data analysis was also performed.

Secondary acute angle closure after lens subluxation is often misdiagnosed as primary acute angle closure (PAAC). The treatment methods for the two are completely different. If there is no preoperative treatment plan for lens subluxation, serious intraoperative and postoperative complications may result. Luo et al. [34] studied 526 eyes with AAC retrospectively, among which 31 eyes (5.89%) were secondary to lens subluxation (misdiagnosed as PAAC). It was found that the main cause of misdiagnosis was neglecting the history of ocular trauma and collection of ocular signs, and lens extraction was an effective surgical method. We studied 160 eyes with acute angle closure, among which 29 eyes (18.13%) were screened with lens subluxation (preoperative mydriasis and clear diagnosis under direct vision), and the incidence rate was significantly higher than that in the above study.

In our study, the clinical onset time in the lens subluxation group was significantly shorter than that in the non-lens subluxation group. Analysis showed that the cause might be that lens subluxation led to AAC, elevated intraocular pressure, rapid disease onset, and obvious pain symptoms. Furthermore, lens subluxation led to lens tilting, uneven tilting of the equator, which stimulated the ciliary body, causing severe ciliary pain, thus shortening the time before seeking medical attention.

Imaging of lens subluxation (UBM and anterior segment OCT) cannot clearly visualize the morphology of the zonule in all quadrants and the scope of rupture. Dislocation of lens can only be indirectly inferred from the distance from the equator of the lens to the ciliary process [7, 18, 35]. However, due to limited detection depth, blurred development of the equator of lens occurs from time to time. It is very important to identify a sensitive parameter for screening of lens subluxation that is relatively clear in UBM imaging, with judgment relatively easy to standardize and quantifiable. Clinical observation revealed that, in AAC eyes with lens subluxation, the proportion of ILA disappearing was relatively high according to quantitative data of UBM image analysis. Moreover, it was found that the proportion of morphological abnormalities of the iris in qualitative data was relatively high (the morphology of nonforward convexity of iris is common). Therefore, this study focused on the two indexes and compared their proportional distribution in the eyes and quadrants with lens subluxation and zonular rupture.

Lens subluxation is always accompanied by zonular rupture. When zonular rupture occurs, the lens is displaced towards the ciliary body and the iris root in the quadrant on the normal side of the zonule (strong traction leads to uneven stress on the lens), the contact area between the anterior surface of the lens and the posterior surface of the iris increases (ILCD becomes longer), the iris lens angle disappears (ILA becomes smaller) (Table 4), and the iris loses its normal convexity (becomes an iris morphology of nonforward convexity) due to the extrusion of the lens (Table 6). Moreover, due to the difference of attachment positions of the zonule and ciliary process as well as ciliary body pronation and supination positions, the lens in the quadrants on normal side may be slightly tilted upwards (attachment point is forward with ciliary body pronation) or slightly tilted downwards (attachment point is backward with ciliary body supination) (Figures 6 and 7). The equator of the lens in the other quadrants deviates from dislocation, and the lower part of the iris in the central part is empty, resulting in an increased iris lens angle, and the whole lens shows “seesaw”-like changes. In this study, the ILA of the case group was smaller than that of the control group in each quadrant; however, the maximum difference values of ILA in each quadrant were significantly larger in the case group than that in the control group, which can be explained by the “seesaw” lens changes in the eyes with lens subluxation (Table 2).

Table 6 shows that the iris in the quadrant has a nonforward convexity structure, and the possibility and risk of zonular rupture are small; therefore, the risk of zonular rupture in the opposite or bilateral quadrants is increased accordingly.

Figures 8 and 9 show that ILA and ILCD in case group and control group had a significant negative correlation. This is consistent with the contact angle between the lens and iris. Furthermore, the lens position in the case group was abnormal, and the proportion of deviation and tilt was high; therefore, the negative correlation between ILA and ILCD was stronger.

Previous studies have mainly focused on the following aspects to judge lens dislocation. (1) UBM was used to measure the depth of the central and peripheral anterior chamber and to compare the differences [36]. However, it does not have specific diagnostic requirements for lens subluxation [37]. (2) The degree and scope of relaxation and rupture of zonule were judged according to the stability of the lens and anterior chamber during cataract surgery [38]. However, the judgment basis is closely related to the operator's proficiency and subjective consciousness; therefore, the credibility is not high. (3) Through UBM image analysis, the difference in distance from the equator of the lens to the ciliary process in each quadrant can be used to indirectly infer lens dislocation [35]. Due to the low resolution of UBM in posterior chamber images, the relevant structures cannot be clearly developed, and only the subjective guess and judgment of the observer can be relied upon. The authenticity and credibility of the data are not high.

In our study, ILA=0 and nonforward convexity of iris were used as sensitive and characteristic indexes of UBM images for screening lens subluxation and zonular rupture for the first time. Data analysis and result judgment can be clearly and intuitively made. Combined with other diagnostic basis and clinical signs, new ideas and hints can be provided for clinical diagnosis of acute angle closure secondary to lens subluxation, and prediction and guiding significance can be provided for selection of surgical approaches and methods for AAC.

## Figures and Tables

**Table 1:** Baseline demographics and clinical data of AAC eyes according to differences of lens position.

Variable	Lens subluxation (29)	Non-lens subluxation (131)	*P*	Total
Sex (F/M)n	23/6	110/21	0.544	133/27
Sex (F/M)%	79.31/20.69	83.97/16.03	0.544	83.12/16.88
Age±*s* (years)	68.55±7.85	66.09±8.52	0.156	66.54±8.44
OD/OS	14/15	65/66	0.896	79/81
Attack time±*s* (days)	1.25±1.08	2.65±2.23	0.028	2.45±2.29
IOP±*s* (mm Hg)	37.10±17.33	34.91±15.73	0.505	35.31±15.99
BCVA±*s* (Decimal)	0.17±0.26	0.22±0.26	0.420	0.21±0.26
Sphere±*s* (Diopter)	0.82±2.02	0.83±2.00	0.832	0.84±2.06
Cylinder±*s* (Diopter)	-0.17±1.10	-0.18±1.06	0.945	-0.17±1.12

s: standard deviation; *P* value indicates the level of significance in the comparison between LS and NLS.

**Table 2:** Comparisons of the quantitative data of UBM images in AAC eyes with lens subluxation and non-lens subluxation ($\overset{-}{x}\pm s$) (*n*).

	Dates from UBM images			
	Lens subluxation	Non-lens subluxation	*P*	95%CI
AOD500±*s*(*n*)				
Superior	0.01±0.02(29)	0.01±0.09(131)	0.662	-0.02~0.01
Inferior	0.02±0.05(29)	0.01±0.04(131)	0.282	-0.01~0.03
Nasal	0.03±0.06(29)	0.02±0.06(131)	0.890	-0.02~0.03
Temporal	0.05±0.08(29)	0.04±0.08(131)	0.638	-0.03~0.04
Whole quadrant	0.03±0.06(116)	0.02±0.06(524)	0.502	-0.01~0.02
Max-Min	0.06±0.08(29)	0.05±0.08(131)	0.428	-0.02~0.05
TIA±*s*(*n*)				
Superior	0.54±1.44(29)	0.72±2.77(131)	0.735	-1.23~0.87
Inferior	1.92±4.57(29)	1.06±3.54(131)	0.263	-0.65~2.38
Nasal	2.04±4.69(29)	1.95±4.97(131)	0.923	-1.90~2.09
Temporal	4.24±6.99(29)	3.81±6.88(131)	0.761	-2.37~3.23
Whole quadrant	2.19±4.96(116)	1.89±4.94(524)	0.551	-0.69~1.30
Max-Min	4.87±6.71(29)	4.00±6.54(131)	0.522	-1.80~3.53
IC±*s*(*n*)				
Superior	0.24±0.16(22)	0.21±0.14(129)	0.447	-0.04~0.09
Inferior	0.25±0.19(28)	0.24±0.14(129)	0.726	-0.05~0.07
Nasal	0.16±0.17(21)	0.16±0.10(131)	0.953	-0.05~0.05
Temporal	0.26±0.12(27)	0.23±0.13(131)	0.296	-0.03~0.08
Whole quadrant	0.23±0.16(98)	0.21±0.13(520)	0.196	-0.01~0.05
Max-Min	0.18±0.14(29)	0.16±0.10(131)	0.396	-0.03~0.06
IS±*s*(*n*)				
Superior	3.57±0.72(22)	3.45±0.67(129)	0.478	-0.20~0.42
Inferior	3.78±1.10(28)	3.52±0.69(129)	0.115	-0.06~0.58
Nasal	3.19±0.56(21)	3.05±0.58(131)	0.298	-0.13~0.41
Temporal	3.37±0.51(27)	3.30±0.66(131)	0.585	-0.19~0.34
Whole quadrant	3.49±0.80(98)	3.33±0.67(520)	0.033	0.01~0.31
Max-Min	0.98±0.86(29)	0.85±0.42(131)	0.253	-0.09~0.34
ILA±*s*(*n*)				
Superior	10.84±10.17(29)	16.63±6.67(131)	0.001*∗*	-8.79~-2.78
Inferior	11.87±8.34(29)	17.18±7.32(131)	0.001	-8.36~-2.27
Nasal	7.40±9.24(29)	14.81±5.04(131)	0.001*∗*	-9.85~-4.98
Temporal	14.32±8.56(29)	18.02±7.05(131)	0.015	-6.67~-0.72
Whole quadrant	9.38±9.37(116)	16.66±6.67(524)	0.001*∗*	-8.74~-5.83
Max-Min	18.58±8.21(29)	11.28±5.84(131)	0.001*∗*	4.74~9.86
ILCD±*s*(*n*)				
Superior	0.95±0.46(22)	0.85±0.39(129)	0.301	-0.09~0.28
Inferior	1.08±0.63(28)	0.91±0.55(129)	0.157	-0.07~0.40
Nasal	1.25±0.65(21)	0.95±0.51(131)	0.017	0.06~0.54
Temporal	1.06±0.71(27)	0.88±0.45(131)	0.091	-0.03~0.39
Whole quadrant	1.08±0.63(98)	0.90±0.48(520)	0.001	0.07~0.29
Max-Min	0.83±0.68(29)	0.66±0.47(131)	0.115	-0.04~0.38
ICPA±*s*(*n*)				
Superior	42.69±25.54(29)	35.82±26.23(131)	0.202	-3.72~17.45
Inferior	50.82±22.64(29)	41.56±22.17(131)	0.044	0.25~18.29
Nasal	47.87±33.63(29)	43.69±30.03(131)	0.508	-8.26~16.63
Temporal	44.92±29.21(29)	45.33±29.73(131)	0.945	-12.44~11.59
Whole quadrant	47.71±25.56(116)	41.60±27.39(524)	0.139	-1.34~9.56
Max-Min	51.32±23.05(29)	45.10±23.36(131)	0.195	-3.22~15.67
LCBA±*s*(*n*)				
Superior	39.23±10.48(29)	41.14±11.69(131)	0.421	-6.56~2.75
Inferior	44.91±10.66(29)	47.47±11.27(131)	0.266	-7.09~1.97
Nasal	51.71±18.35(29)	51.60±13.64(131)	0.973	-5.81~6.02
Temporal	49.02±12.46(29)	51.21±13.65(131)	0.430	-7.63~3.27
Whole quadrant	46.22±14.02(116)	47.85±13.26(524)	0.235	-4.34~1.06
Max-Min	25.00±13.49(29)	24.49±11.94(131)	0.839	-4.45~5.47
AL ±*s*(*n*)	22.38±0.83(29)	24.49±11.94(131)	0.966	-0.32~0.34
CACD ±*s*(*n*)	2.33±0.32(29)	2.38±0.27(131)	0.393	-0.16~0.06
LT ±*s*(*n*)	4.97±0.37(29)	4.71±0.60(131)	0.022	0.04~0.50
LAF ±*s*(*n*)	2.22±0.17(29)	2.11±0.29(131)	0.041	0.00~0.23
LP ±*s*(*n*)	4.82±0.36(29)	4.74±0.27(131)	0.149	-0.03~0.20
RLP ±*s*(*n*)	2.15±0.16(29)	2.12±0.13(131)	0.183	-0.02~0.09

s: standard deviation; *∗P*<0.001.

**Table 3:** Percentage of lens subluxation in AAC eyes with or without ILA=0.

AAC eyes			
Eyes (*n*)	Lens subluxation (*n*)	Non-lens subluxation (*n*)	Total (*n*)
ILA=0 in one quadrant at least	22	5	27
ILA=0 in no quadrant	7	126	133
Total	29	131	160

Chi-square test: *X*^*2*^=87.859, *P*<0.001; OR=79.200 (95%CI 23.063~271.983); OR: odds ratio.

**Table 4:** Percentage of zonular rupture in the quadrants with or without ILA=0 in AAC eyes.

AAC eyes			
Quadrants (*n*)	Zonular rupture (*n*)	Non-zonular rupture (*n*)	Total (*n*)
Quadrants with ILA=0	10	27	37
Quadrants without ILA=0	26	577	603
Total	36	604	640

Chi-square test: *X*^*2*^=33.884, *P*<0.001; OR=0.122 (95%CI 0.053~0.278); OR: odds ratio.

**Table 5:** Percentage of lens subluxation in AAC eyes with NFCI in one quadrant at least or FCI in all quadrants.

AAC eyes			
Eyes (*n*)	Lens subluxation (*n*)	Non-lens subluxation (*n*)	Total (*n*)
NFCI in one quadrant at least	17	67	84
FCI in all quadrants	10	66	76
Total	27	133	160

Chi-square test: *X*^*2*^=1.426, *P*=0.232; OR=0.597 (95%CI 0.255~1.400); OR: odds ratio; NFCI: nonforward convexity of iris; FCI: forward convexity of iris.

**Table 6:** Percentage of zonular rupture in quadrants with NFCI or FCI in AAC eyes.

AAC eyes			
Quadrants (*n*)	Zonular rupture (*n*)	Non-zonular rupture (*n*)	Total (*n*)
Quadrants with NFCI	12	136	148
Quadrants with FCI	16	476	492
Total	28	612	640

Chi-square test: *X*^*2*^=6.413, *P*=0.011; OR=0.381 (95%CI 0.176~0.825); OR: odds ratio; NFCI; nonforward convexity of iris; FCI: forward convexity of iris.

## Data Availability

The data used to support the findings of this study are available from the corresponding author upon request.

## Sketch for All the Parameters in the Study

AOD 500: Angle opening distance at 500*μ*m from scleral spur
TIA: Trabecular iris angle
ACA 500: Anterior chamber angle at 500*μ*m from scleral spur
IC: Iris convexity
IS: Iris span
ILA: Iris lens angle
ILCD: Iris lens contact distance
ICPA: Iris ciliary processes angle
LCBA: Limbus ciliary body angle
AL: Axial length
CACD: Central anterior chamber depth
LT: Lens thickness
LAF: Lens-axial length factor
LP: Lens position
RLP: Relative lens position.
